# Differences in Muscle Oxygenation, Perceived Fatigue and Recovery between Long-Track and Short-Track Speed Skating

**DOI:** 10.3389/fphys.2016.00619

**Published:** 2016-12-15

**Authors:** Florentina J. Hettinga, Marco J. Konings, Chris E. Cooper

**Affiliations:** Centre for Sports and Exercise Science, School of Biological Sciences, University of EssexColchester, Essex, UK

**Keywords:** near-infrared spectroscopy, elite athletes, training, endurance, winter sport

## Abstract

Due to the technical nature of speed skating, that is affecting physiological mechanisms such as oxygenation and blood flow, this sport provides a unique setting allowing us to uncover novel mechanistic insights of the physiological response to exercise in elite middle-distance and endurance sports. The present study aimed to examine the influence of skating mode (short-track vs. long-track) on muscle oxygenation, perceived fatigue, and recovery in elite speed skating. Muscle oxygenation of 12 talented short-track speed skaters was continuously monitored during a long-track (LT) and a short-track (ST) skating time-trial of maximal effort using near-infrared spectroscopy (NIRS) on the m. vastus lateralis for both legs. Video captures were made of each testing session for further interpretation of the muscle oxygenation. To determine recovery, perceived exertion was measured 2 and 4 h after each testing sessions. Repeated measures ANOVA's were used for statistical analysis (*p* < 0.05). After a rapid desaturation in both legs directly after the start, an asymmetry in muscle oxygenation between both legs was found during LT (tissue saturation-index (TSI%)-slope: left = 0.053 ± 0.032; right = 0.023 ± 0.020, *p* < 0.05) and ST speed skating (TSI%-slope: left = 0.050 ± 0.052, right = 0.001 ± 0.053, *p* < 0.05). Resaturation of the right leg was relatively lower in ST compared to LT. For the left leg, no difference was found between skating modes in muscle oxygenation. Respectively, two (*ST* = 5.8 ± 2.0; *LT* = 4.2 ± 1.5) and 4 h (*ST* = 4.6 ± 1.9; *LT* = 3.1 ± 1.6) after the time-trials, a higher rate of perceived exertion was found for ST. Based on our results, ST seems more physiologically demanding, and longer periods of recovery are needed after training compared to LT. Technical aspects unique to the exercise mode seem to impact on oxygenation, affecting processes related to the regulation of exercise intensity such as fatigue and recovery.

## Introduction

Speed skating is an intriguing sport to study, in particular because speed skaters need to adopt the biomechanically favorable crouched position that is essential for speed skating performance (Van Ingen Schenau et al., 1983; Konings et al., 2015). This crouched position combined with a relatively long gliding phase and high intramuscular forces at the same time leads to a physiological disadvantage as it is suggested to increase deoxygenation of the working muscles (Rundell, 1996; Rundell et al., 1997; Konings et al., 2015). The presence and magnitude of deoxygenation during speed skating has been investigated using near infrared spectroscopy, demonstrating a linear relationship with knee-angle in long track speed skating (Foster et al., 1999) and an asymmetry between the legs in short track skating, particularly related to cornering (Hesford et al., 2012, 2013a,b). Most likely, the reduced blood flow to the working muscles reduces the aerobic capacity of the recruited muscle groups (Rundell, 1996), whereas not all the available oxygen can be transported toward the working muscles during speed skating. The reduced blood flow is expected to exacerbate processes related to perceived fatigue and recovery, thereby impacting on elite performance. Due to the technical nature of speed skating that is affecting physiological mechanisms such as oxygenation and blood flow, this sport provides a unique setting allowing us to uncover novel mechanistic insights into these aspects of the physiological response to exercise in elite sports.

Changes in muscle oxygenation can inform on a drop in tissue pO_2_ and therefore potentially correlate with fatigue and performance. Global hypoxia decreases arterial oxygenation and can exacerbate peripheral fatigue and fatigability (Katayama et al., 2007; Romer et al., 2007). Local ischemia leading to a 7% or larger reduction in muscle oxygenation can lead to decreased muscle production in the fore-arm muscles (Murthy et al., 2001). Increased intramuscular pressure has been suggested to decrease blood flow and thereby reduce tissue oxygenation at isometric contractions >25–35% of maximal torque capacity in upper leg muscles (de Ruiter et al., 2007). Under these conditions the muscle fibers will largely depend on glycolysis as an energy source, potentially exacerbating fatigue.

Long track and short track speed skating are expected to differ in terms of asymmetry between the legs, associated with the technical nature and characteristics of the different sports. In our communication with coaches and athletes, we became aware that when imposing testing sessions of similar load in short-track (ST) and long-track (LT) skating to elite ST athletes, the athletes felt more fatigued after ST compared to LT skating sessions. As short track athletes compete on a shorter track with shorter straights and more and tighter curves during their race, differences in intra-muscular pressure, aerobic physiology and thereby experienced fatigue and recovery could be expected.

Therefore, the aim of the current study was to measure the influence of skating mode (short-track vs. long-track) on muscle oxygenation, and determine how this affects perceived fatigue and recovery in speed skating. The crouched position of speed skaters leads to a unique form of physiological stress: static occlusion of blood flow that occurs repetitively with every cycle, that might hinder recovery related to oxygenation (Rundell, 1996; Rundell et al., 1997; Foster et al., 1999). In short track speed skating, with tighter corners, longer periods of occlusion are seen for the right leg in particular (Hesford et al., 2012, 2013a,b), and we expect this will impact on recovery and perceived fatigue. It is hypothesized that the working muscles will remain more deoxygenated during short-track speed skating, associated with the relatively long periods of static contraction, and that there is thereby a larger asymmetry between the left and right leg. It is expected that this will lead to higher experienced fatigue, and longer periods of recovery after short-track speed skating compared to long-track speed skating. If a link between muscle oxygenation and fatigue is clear for the same elite athletes in a single sport (speed skating) depending only on the mode of the exercise, this suggests that measures of muscle oxygenation could inform in constructing training and recovery loads in other sports or activities involving local ischemia, even ones that do not have a specific asymmetric component.

## Materials and methods

### Participants

Twelve talented elite short-track speed skaters (11 males, 1 female) from the Dutch national U-23 short-track speed skating team (age = 19,7 ± 2,6 year, height = 179,8 ± 6,5 cm, mass = 71,0 ± 6,4 kg) participated in this study. All were also familiar with long-track speed skating, in which they engaged on a weekly basis as part of their training regime. All participants, and also their parents if they were <18 years, gave their written informed consent before participation. This study was approved by the local ethics committee (University of Essex, UK) and in accordance with the Declaration of Helsinki.

### Experimental procedures

Testing took place on a long-track (400 m) and short-track (111.12 m) speed skating oval both approved for international competition and at the same venue. Participants was asked to complete two testing conditions: a short-track time trial and a long-track time trial of comparable intensity, counter-balanced with a time interval of a week separating the two trials. All measurements took place on Tuesday mornings (08 h 30–10 h 30). The order of the testing conditions was counterbalanced between participants and separated by 1 week for each participant. Before each testing condition, participants performed their own warm-up, as they would when preparing for a normal training session.

In each testing condition participants had to complete a time-trial with flying start as fast as possible, after a warm-up designed by the coach. Warm-up and time-trial were separated by 10 min of active rest. All time trials were completed individually. In one testing condition the time-trials involved two laps on a long-track oval (800 m), in the other six laps on a short-track oval (total trial length: 666 m). All the testing sessions were set up in cooperation with the Dutch national short-track coaches. Long-track and short-track testing sessions were set up in such a way that they were of an equal expected physiological intensity (i.e., maximal) to make the different skating modes comparable, whilst interfering as little as possible with the coach and the usual training set-up to ensure an high ecological validity and practical relevance.

The muscle oxygenation of both legs was monitored continuously using NIRS during all time-trials. To quantify the physiological intensity of the time-trials heart rate was measured during the time-trials, in combination with the mean lactate values and rate of perceived exertion directly after the time-trials. Furthermore, mean duration, velocity, acceleration, and VO_2_ were determined during the time-trial in order to assess if they were related to the oxygenation at local muscular level. Lastly, two relevant technical variables for speed skating, the knee- and trunk angle respectively, were determined during the time-trials, as the knee angle was found to be related with the extent of deoxygenation in speed skating (Foster et al., 1999).

To determine perceived fatigue and recovery, first of all, rates of experienced exertion were collected after each testing session, as well as after 2 h and 4 h after completion of the testing condition. Secondly, before and after each testing condition the participants performed a 6-s maximal cycle test followed by 30-s of rest. The first 6-s maximal cycle test was completed before the warm-up. The time-interval between completion of the testing condition and the second 6-s maximal cycle test was controlled for both trials, and did not exceed 5 min. In this 30-s rest period, recovery in muscle oxygenation for this single sprint was determined in order to quantify their recovery after each testing condition.

### Muscle oxygenation measurements

A wireless spatially resolved spectroscopy (SRS) dual-wavelength oximeter (Portamon; Artinis Medical Systems, BV, The Netherlands) was used to measure absolute and relative changes in NIRS parameters. The Portamon has previously been used in speed skating in order to investigate muscle oxygenation and hemodynamics (Hesford et al., 2012, 2013a,b). The unit is self-contained and compact, measuring 83 × 52 × 20 mm and weighs with battery 84 g. The Portamon has three pairs of light-emitting diodes that emit light of wavelengths 760 and 850 nm and are positioned 30, 35, and 40 mm from the detector. With these wavelengths, changes in concentration of the chromophores hemoglobin (Hb) and myoglobin (Mb) can be detected. Although using a two-wavelength spectrometer allows a measurement of changes in the concentration of oxygenated and deoxygenated species discrimination between the oxygenated and deoxygenated forms of respectively Hb and Mb cannot be made. Therefore, the tissue oxygenation changes reported will contain contributions from both chromophores, although the myoglobin contribution is likely to be smaller (see discussions in Hesford et al., 2012, 2013a,b; Born et al., 2014) and was ignored for the purposes of this discussion.

Single distance continuous wave dual wavelength NIRS can only report on changes in chromophore concentrations. However, multi distance systems such as the Portamon device can take advantage of SRS. SRS enables biasing of the NIRS data away from superficial layers (skin/adipose) layers in favor of deeper (muscle) changes, whilst at the same time providing an absolute measure of tissue oxygen saturation TSI% (Suzuki et al., 1999). During the time-trials, NIRS was used to measure continuously changes in total muscle hemoglobin (tHb), muscle oxyhemoglobin (HbO_2_), muscle deoxyhemoglobin (HHb) and muscle oxygen saturation (TSI%) in both left and right m. vastus lateralis. A baseline was determined based on 30 s averaging before participants went on-ice to start their TT. All chromophore concentration changes were presented relative to this baseline (Jones et al., 2015). Video captures of the testing sessions was used to match NIRS changes with the position and movement of the skaters.

The Portamon devices were placed on the belly of the musculus vastus lateralis on both legs, midway between the lateral femoral epicondyle and the greater trochanter of the femur. The devices were fixed into position using surgical tape, in order to ensure the optodes and detector did not move relative to the participant's skin. Precise and consistent optode placement was crucial, as quadriceps muscle oxygenation was shown to be non-uniform during exercise (Quaresima et al., 2001; Kime et al., 2005; Kennedy et al., 2006). To ensure that the NIRS measurements were not influenced by environmental light, participants wore a black bandage attached over both the Portamon devices. All participants reported that the devices in combination with the bandages did not restrict their movements in any way.

Finally, NIRS was used to examine differences in recovery between both skating modes. To measure differences in recovery after the testing conditions, participants performed a 6-s maximal cycle test before and after each testing session followed by 30-s of rest. The tests took place on a Wattbike Pro (Wattbike Ltd., UK) and participants were allowed to choose their own saddle height before their first test. During both testing conditions, saddle height was kept constant and participants were instructed to stay seated straight on the Wattbike during the 30-s of rest. Peak power output (PPO) was the highest recorded power output during each sprint. Values for ΔTSI% are reported as a change from baseline (5 s averaging before the sprint). The magnitude of the drop in ΔTSI% during the 6-s sprint was determined as the difference in TSI% between baseline and the lowest 0.5-s average during the sprint. Recovery was determined based on the half-time recovery in ΔTSI% after the 6-s sprint. Halftime recovery was defined phenomenologically as the time it took before TSI% recovered to half of the difference between the initial baseline value and the lowest TSI value.

### Rate of perceived exertion measurements

Participants were asked for their rates of perceived exertion (RPE) on a Borg CR10 scale directly after each testing condition (Borg, 1998). To monitor the (experienced) recovery after each testing condition, participants was also asked to report their level of fatigue on a Borg CR10 scale 2 and 4 h after each testing session, respectively. All participants were familiar with using the Borg scale, whereas the Borg scale was already used regularly after testing sessions before the start of the measurements.

### Global physiological measurements

Respiratory gases were measured during each testing session using a wearable and wireless breath-by-breath pulmonary gas analyzer (Metamax 3B, Cortex, Germany) previously demonstrated to be valid and reliable for measuring oxygen uptake (Macfarlane and Wong, 2012). The Metamax 3B was calibrated each day a measurement took place using a Jaeger 3L syringe. For each the test an ambient air measurement was also performed. As the oxygen use and transport adaptations require some time, and thus lead to a lag in the aerobic response (Hagerman, 2000), only the last 30 s of the time-trial were averaged, resulting in a mean oxygen uptake value (VO_2_ mean). Additionally heart rate was monitored during the testing sessions by a validated portable heart rate monitor (Zephyr Bioharness, Zephyr Technology, New Zealand; Franklin and Brooks, 2012).

### Velocity measurements

Position, velocity and acceleration of the participants were monitored in all time-trials by the Local Positioning Measurement (LPM-) system (Inmotio Object Tracking BV, The Netherlands). The LPM-system is a high frequency (1000 Hz), radio-frequency based technology. Participants wore a vest containing a transponder located on the back that was connected to two antennas, one on top of each shoulder. To understand how the skaters' movements affected the differing physiological demands during one lap, positional data collected by the LPM system were synchronized with the data collected by NIRS. For the purpose of this analysis two separate elements (which are all repeated twice per lap) were defined and analyzed:

1. Straight: the section of the lap between the two corners. The straight starts when the left blade touches the ice after the crossover of legs while exiting the corner. The straight contains in short-track speed skating one glide on each blade and ends when the left blade touches the ice after the right foot glide. During long-track speed skating the straight is characterized by several strokes containing a gliding phase, push-off, and repositioning phase (Konings et al., 2015).
2. Corner: the phase between the end of the straight and the moment at which the skater's left blade touches the ice to begin the gliding phase of the straight the right blade touches the ice to begin the hang. This phase contains in short-track speed skating three subsections in the following order: an entry at which the skater performs respectively a series of leg crossovers, a hang in which the skater travels around the apex of the corner supported solely on the right blade, and an exit in which another series of leg crossovers while maintaining a very low position were performed (Hesford et al., 2012). In contrast, in long-track speed skating a series of leg crossovers is conducted when cornering.

### Technical measurements

The trunk- and knee angle are two crucial technical characteristics for speed skating performance (Konings et al., 2015). Participants were filmed at the straights during their testing conditions with one digital high-speed camera to assess their knee and trunk angles. The camera was placed perpendicular to the sagittal plane of the speed skater during all sessions at both speed skating tracks. To determine the trunk- (line between neck-hip and horizontal) and knee angle (between upper and lower leg) the recordings when the subject was in his gliding phase were analyzed.

### Lactate measurements

Within 1 min after completion of the second time-trial, the lactate value of the participants was measured using Lactate Pro (Arkray Inc, Japan) by using capillary blood from the finger after a prick in the finger. Lactate measurements were performed by an experienced test leader.

### Data-analysis and statistics

All statistical analyses were performed using SPSS version 19.0. For statistical analysis the slope in ΔTSI%, ΔHbO_2_, ΔHHb, and ΔtHb over time after the initial desaturation was determined for each subject. Additionally to the NIRS measurements, the positional data and video captures of the testing conditions collected using the LPM-system were used for further detailed analysis of the muscle oxygenation during the testing condition. For all variables, mean and SD were determined and used for further statistical analysis. Statistical comparisons of each measured variable between short-track and long-track skating were made by using repeated measures ANOVA. In addition, comparisons between both legs for each testing condition were performed for ΔTSI%, ΔHbO_2_, ΔHHb, and ΔtHb in order to examine the occurrence of an asymmetry in blood flow to the working muscles. Finally, absolute values of TSI% of both legs in both skating modes were determined during the final three strokes of the time trial. Descriptive statistics are presented as means ± SD unless otherwise noted. The statistical significance was defined at *P* < 0.05.

## Results

**Figure 1:**
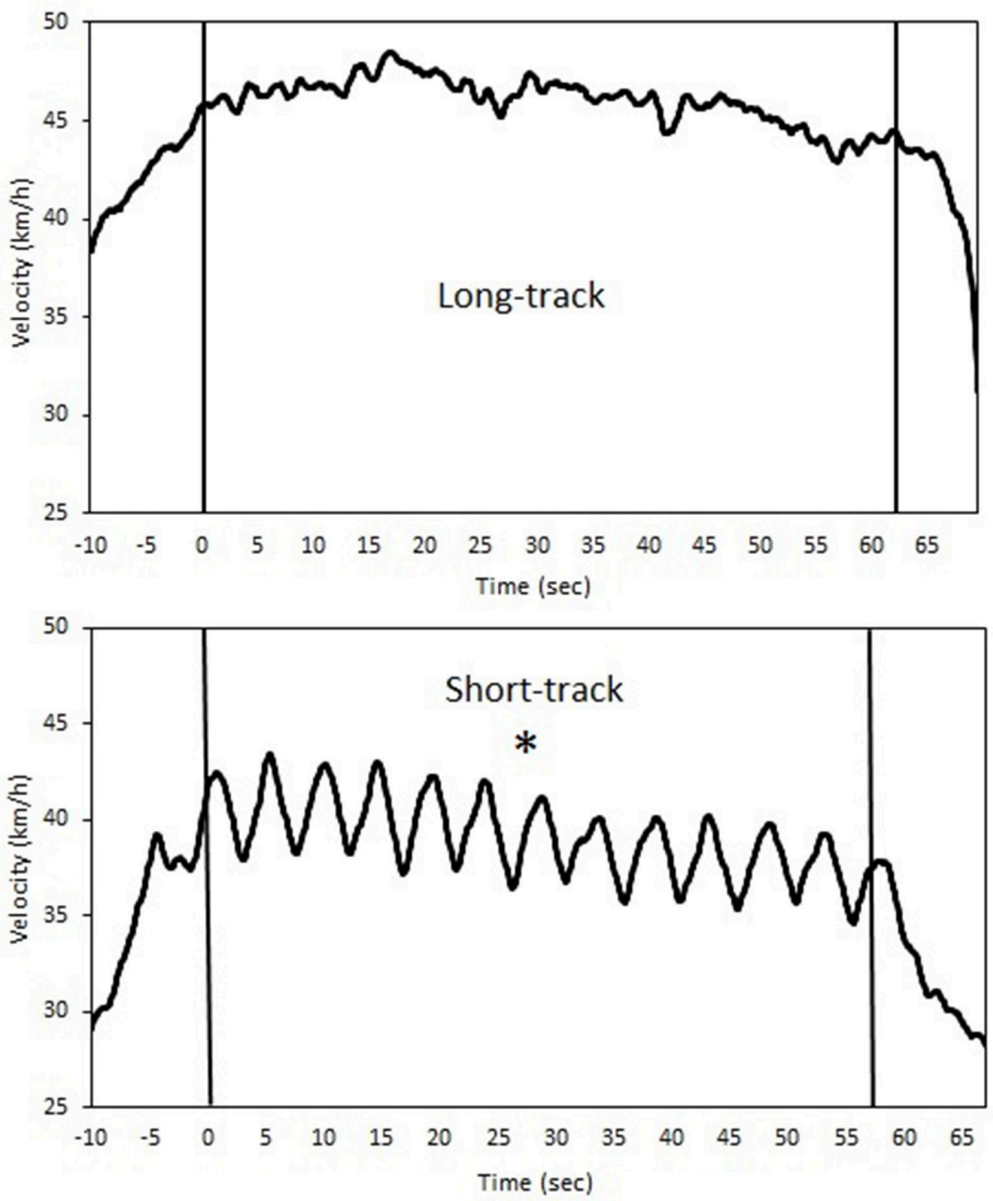
A typical example of the velocity profiles during both long-track (upper panel) as well as short-track (lower panel) speed skating for a single time-trial (^*^indicates a significant difference between skating modes for velocity and acceleration, *p* < 0.05).

Means ± SD of the time-trial performance for the long-track time-trials (LT) and the short-track time-trials (ST) can be found in Table 1. Faster completion times (*LT* = 63.45 ± 2.20, *ST* = 60.94 ± 2.90 s; *p* = 0.005) and a higher mean velocity (*LT* = 44.36 ± 1.97, *ST* = 38.26 ± 1.46 km/h; *p* < 0.001) were found for LT compared to ST. Nevertheless, no differences in heart rate (*LT* = 178.8 ± 5.8, *ST* = 179.6 ± 6.6 bpm; *p* = 0.609), lactate (*LT* = 10.5 ± 1.5, *ST* = 11.0 ± 1.1 mmol/l; *p* = 0.356) or RPE (*LT* = 8.9 ± 1.2, *ST* = 9.2 ± 1.1; *p* = 0.555) were found between the testing conditions, suggesting time-trials could be perceived as of a similar physiological intensity. No differences in knee angle were reported between conditions (*LT* = 103.4 ± 4.5, *ST* = 103.8 ± 2.9°; *P* = 0.843). However, a lower trunk angle was found during ST (*LT* = 16.6 ± 1.1, *ST* = 15.3 ± 0.9°; *p* < 0.001). In addition, deceleration of the participants was higher after ST compared to LT (see Table 1 and Figure 1).

**Table 1:** **Means ± SD of the time-trial characteristics, the absolute TSI% values in the final three strokes of the time-trial and the slope of the recovery in muscle oxygenation throughout the time-trial for both long-track as well as short-track time-trials (*N* = 12)**.

	**Means (*****SD*****)**		***F*-value (sign.)**
	**Long-track**	**Short-track**	
**TIME-TRIAL CHARACTERISTICS**			
Completion time (sec)	63.45 ± 2.90	60.94 ± 2.20	*P* = 0.005^*^
Velocity (km · h^−1^)	44.4 ± 2.0	38.3 ± 1.5	*P* < 0.001^*^
Acceleration (km · min^−2^)	−7.2 ± 2.9	−11.0 ± 2.5	*P* = 0.005^*^
VO_2_mean (ml · kg^−1^ · min^−1^)	59.7 ± 3.6	57.8 ± 3.7	*P* = 0.019^*^
Lactate (mmol · l^−1^)	10.5 ± 1.5	11.0 ± 1.1	*P* = 0.356
Heart rate (min^−1^)	178.8 ± 5.8	179.6 ± 6.6	*P* = 0.609
**ABSOLUTE TSI% IN THE FINAL THREE STROKES OF THE TIME-TRIAL**			
Left leg	51.5 ± 5.9	50.8 ± 5.4	
Right leg	50.2 ± 4.0	46.4 ± 6.7	
Left LT vs. Left ST			*P* = 0.405
Right LT vs. Right ST			*P* = 0.028^*^
Left ST vs. Right ST			*P* = 0.005†
Left LT vs. Right LT			*P* = 0.358
**SLOPE OF THE MUSCLE OXYGENATION RECOVERY DURING**			
**TIME-TRIALS ΔTSI% Slope (ΔTSI% · min^−1^)**			
Left leg	5.3 ± 3.2	5.0 ± 5.2	
Right leg	2.3 ± 2.0	0.1 ± 5.3	
Left LT vs. Left ST			*P* = 0.825
Right LT vs. Right ST			*P* = 0.049^*^
Left ST vs. Right ST			*P* = 0.001†
Left LT vs. Right LT			*P* < 0.001†
**ΔHbO_2_ Slope (μM · cm · min^−1^)**			
Left leg	227.2 ± 78.6	165.5 ± 35.5	
Right leg	200.1 ± 117.6	112.8 ± 61.6	
Left LT vs. Left ST			*P* = 0.489
Right LT vs. Right ST			*P* = 0.004^*^
Left ST vs. Right ST			*P* = 0.017†
Left LT vs. Right LT			*P* = 0.507
**ΔHHb Slope (μM · cm · min^−1^)**			
Left leg	5.6 ± 21.8	−4.9 ± 38.0	
Right leg	−13.1 ± 10.3	6.8 ± 45.1	
Left LT vs. Left ST			*P* = 0.540
Right LT vs. Right ST			*P* = 0.151
Left ST vs. Right ST			*P* = 0.493
Left LT vs. Right LT			*P* = 0.087
**ΔtHb Slope (μM · cm · min^−1^)**			
Left leg	102.8 ± 59.7	80.3 ± 26.4	
Right leg	107.0 ± 39.4	59.8 ± 15.1	
Left LT vs. Left ST			*P* = 0.407
Right LT vs. Right ST			*P* = 0.008^*^
Left ST vs. Right ST			*P* = 0.012†
Left LT vs. Right LT			*P* = 0.842

* Significant difference between skating mode (p < 0.05)

† *Significant difference between both legs (p < 0.05)*.

**Figure 2:**
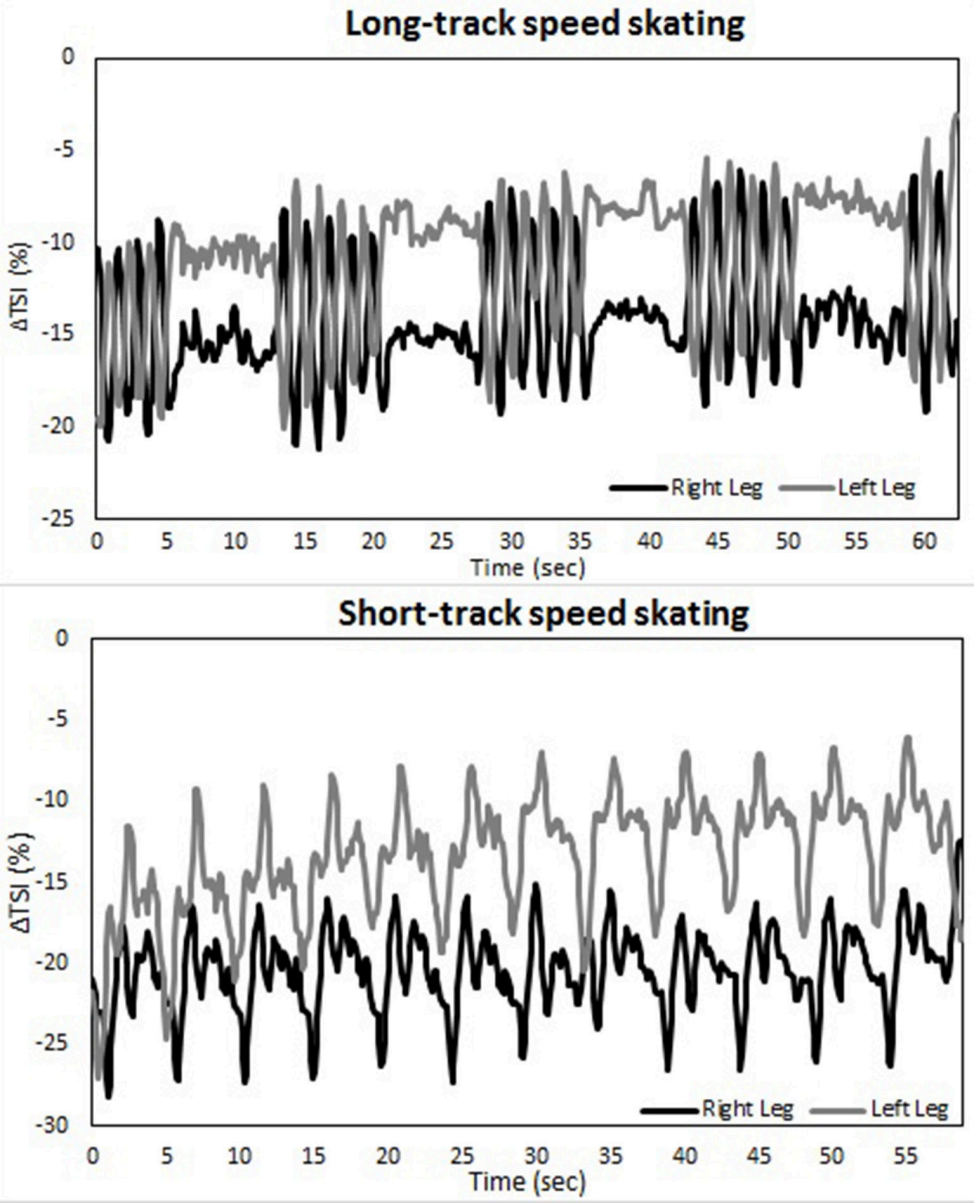
**A typical example of the Δ tissue saturation index (%) during both a long-track (upper panel) as well as a short-track (lower panel) time-trial for one subject**.

**Figure 3:**
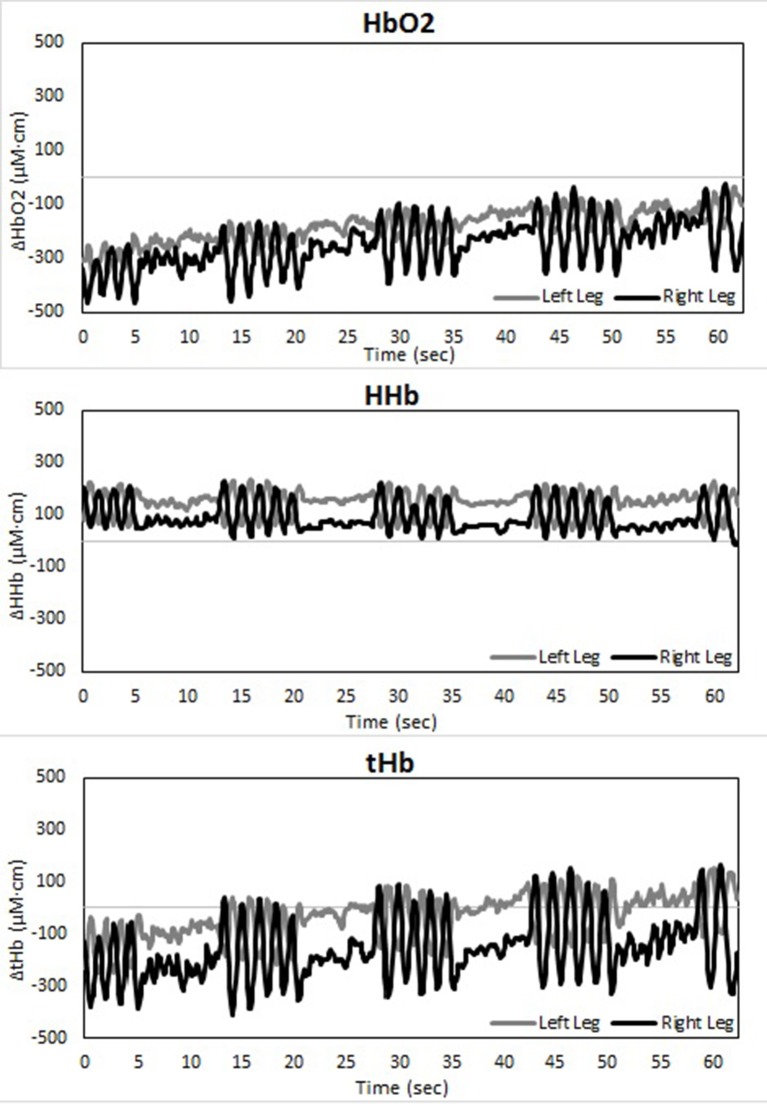
**Temporal changes in right and left vastus lateralis during the long-track time trial: HbO_2_ (upper panel), HHb (middle panel), and tHb (lower panel)**.

**Figure 4:**
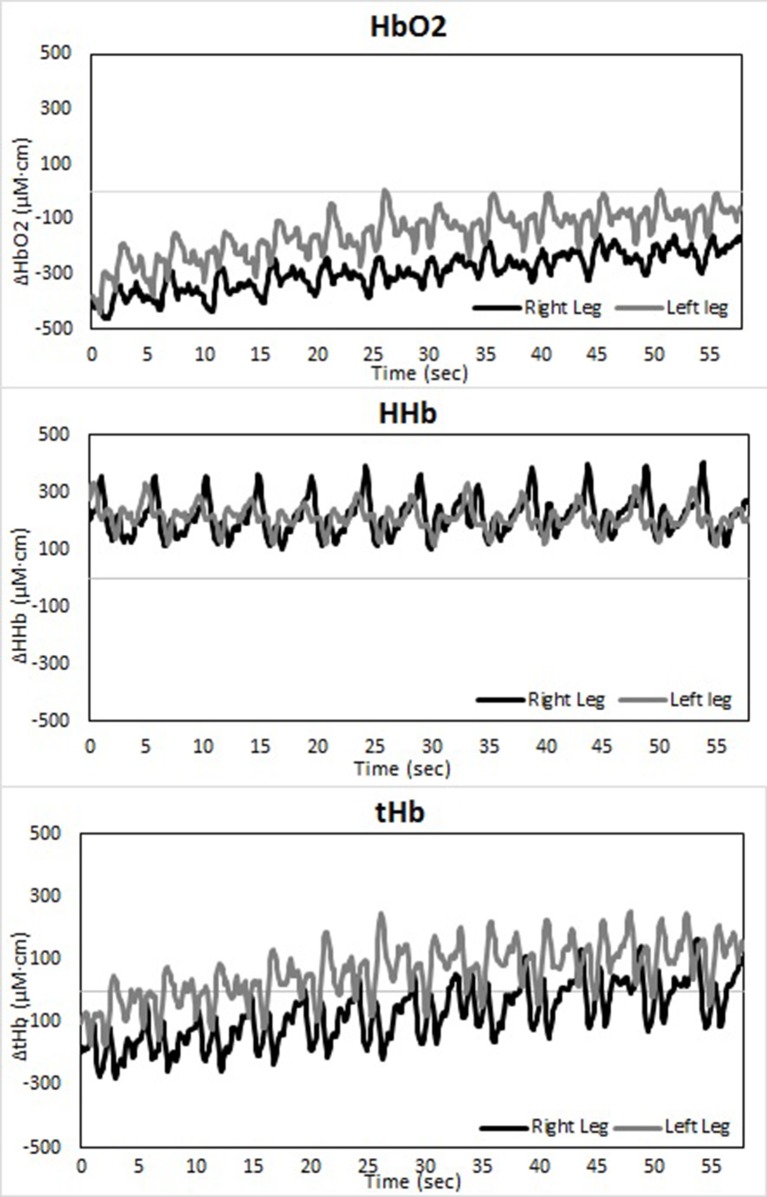
**Temporal changes in right and left vastus lateralis during the short-track time trial: HbO_2_ (upper panel), HHb (middle panel), and tHb (lower panel)**.

Changes in muscle oxygenation between both legs occurred in both LT as well as ST (see Table 1 and Figures 2–4). Typical NIRS traces are shown for TSI (Figure 2) and HHb, HbO_2_, and tHb (Figures 3, 4). They illustrate that at the start of the race compared to baseline there is a fall in muscle oxygen saturation (TSI) caused by rise in HHb and fall in HbO_2_. The fall in HbO_2_ is larger than the rise in HHb, resulting in a consequent fall in tHb. As the TT progresses, muscle oxygenation (TSI) increases. This is caused entirely by an increase in HbO_2_. There is no change in HHb, with the result that tHb increases along with HbO_2_.

There is an asymmetry in muscle oxygenation between left and right legs as the TT progresses. Table 1 shows the rate of change in time for the NIRS parameters. Muscle oxygenation significantly increases in the left leg compared to the right in both ST and LT. However, the difference is larger in ST than LT. These oxygenation changes are dominated by HbO_2_ and not HHb. HbO_2_ increases in both LT and ST with the larger differences being seen in ST. There is no significant change in HHb in either leg. An HbO_2_ increase with no change in HHb is reflected in an increase in tHb. Again this increase is larger for the left leg than the right leg in ST compared to LT.

The use of spatially resolved methods enabled an absolute measurement of muscle oxygen saturation (TSI) at the end of the TT. The only difference between legs was seen for the right leg in ST. Muscle oxygenation in left ST, right ST and left ST were equivalent to one another and all higher than right ST.

**Figure 5:**
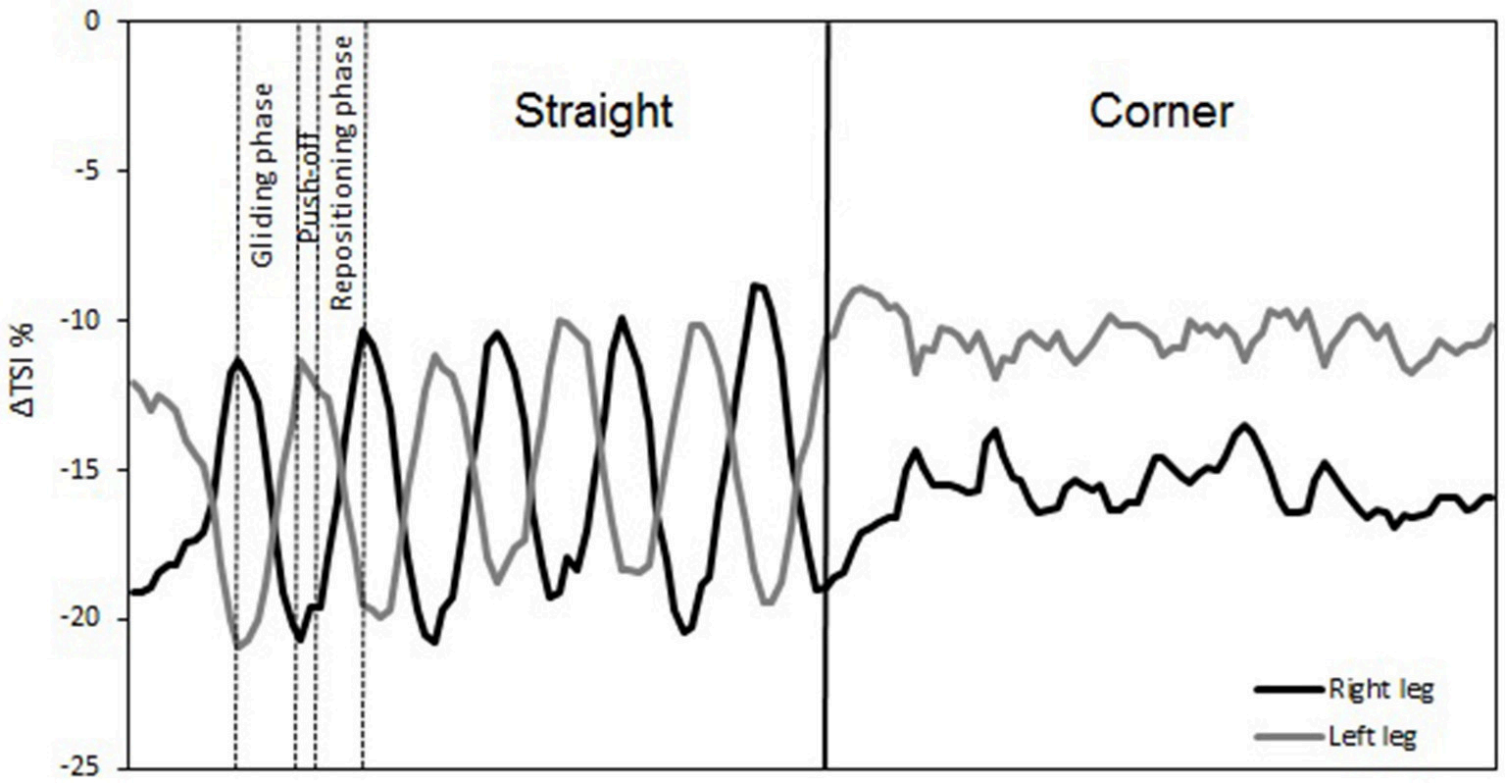
**Δ Tissue saturation index (%) in one lap during long-track speed skating**. Straights in long-track speed skating are characterized by a repetitive cyclic pattern of three phases per leg: gliding phase, push-off (leg on-ice), and repositioning phase (leg off-ice). These three phases are remarked for a single stroke of the right leg. The corners are characterized by a series of leg crossovers.

**Figure 6:**
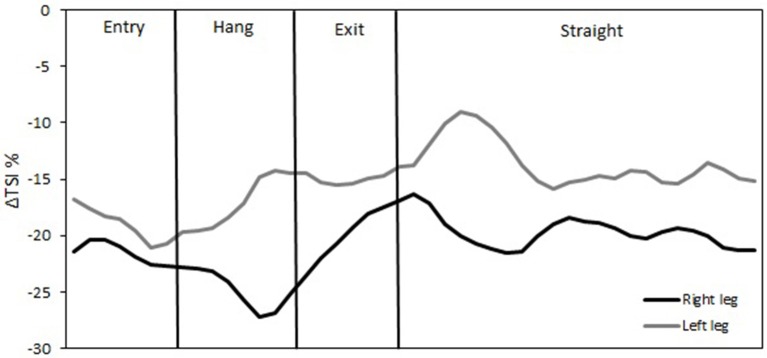
**Δ Tissue saturation index (%) in one lap during short-track speed skating**. The straight in short-track speed skating contains one glide on each blade (both legs on-ice). The corner in short-track speed skating contains three subsections in the following order: an entry at which the skater performs leg crossovers, a hang in which the skater travels around the apex of the corner supported solely on the right blade, and an exit involving leg crossovers.

**Figure 7:**
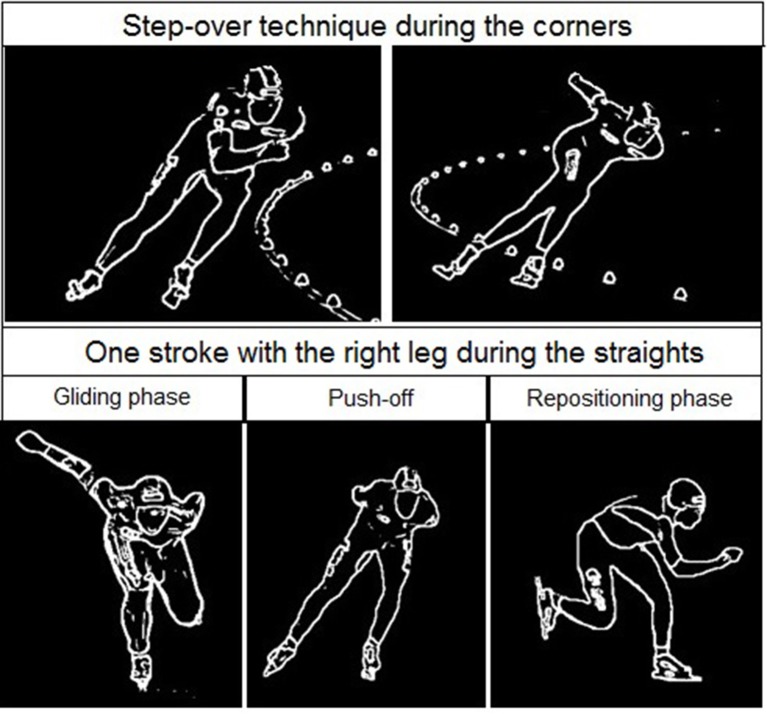
**Skating at the corner (upper panel) and the straight (lower panel) in long track speed skating**. The corner typically contains a series of leg crossovers. The straight is characterized by several strokes containing a gliding phase (left panel), push-off (middle panel), and repositioning phase (right panel) as shown in the lower figure for the right leg.

**Figure 8:**
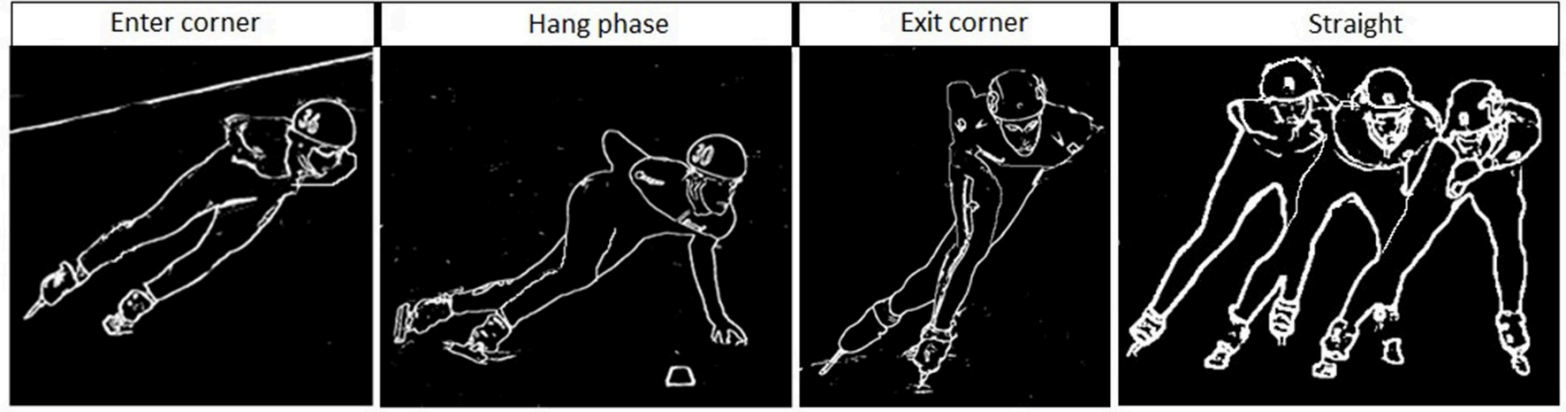
**Skating at the corner (first three panels) and the straight (right panel) in short track speed skating**. The corner in short-track speed skating contains three subsections in the following order: an entry at which the skater performs leg crossovers, a hang in which the skater travels around the apex of the corner supported solely on the right blade, and an exit involving leg crossovers. The straight contains in short-track speed skating a glide on each blade and ends when the left blade touches the ice after the right foot glide.

The temporal changes in TSI, tHb, HbO_2_, and HHb reveal significant differences in oxygenation in the course of a single lap. This can be illustrated by higher resolution analysis of a single half lap in LT (Figure 5) and ST (Figure 6). The lap was divided into segments indicated in both figures. An illustration of how these different segments relate to technique can be found in Figure 7 (LT) and Figure 8 (ST) During the straights in LT (Figure 5) the leg desaturates in the gliding phase and push-off (when the leg is on the ice), while the leg resaturates in the repositioning phase (when the leg is off the ice). In ST (Figure 6), the typical way of traveling (hanging) around the corner on only the right leg leads to a resaturation of the left leg and a desaturation of the right leg.

**Figure 9:**
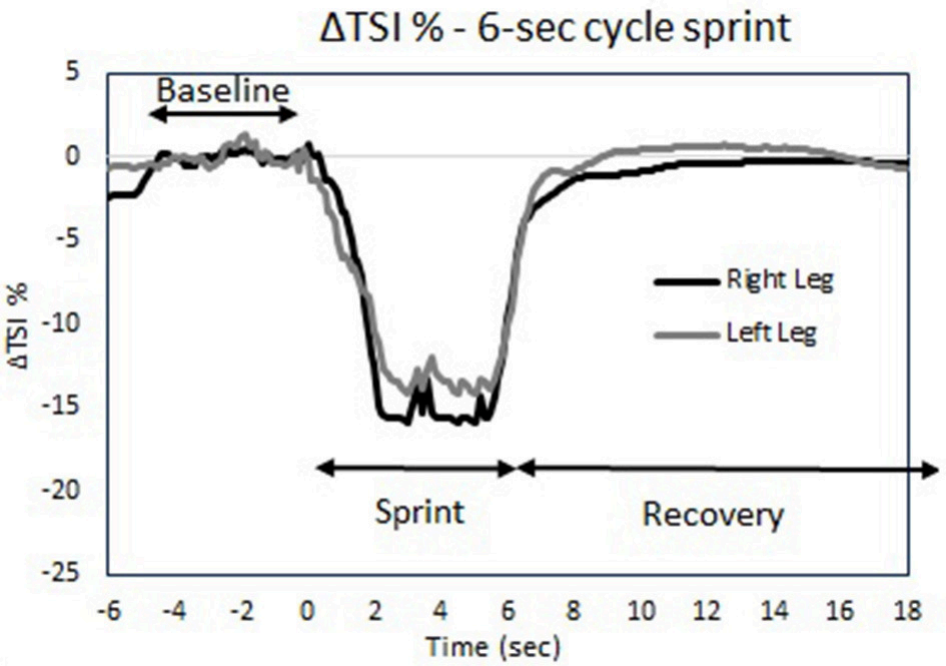
**Tissue saturation index trace (ΔTSI%) during the 6-s sprint cycle test protocol for both legs**.

Figure 9 indicates the changes in muscle oxygenation during a 6-s sprint cycling test taken before and after the time trials. No differences were found in the drop of the ΔTSI% during the 6-s sprint between the legs, between the pre and post-test or between skating modes (*P* > 0.05; Table 2). The recovery halftime in ΔTSI% after the post 6-s sprint test was significantly increased after ST compared to LT. However, there were no differences in recovery rates between the legs for either skating mode (see Table 2). In addition, participants reported they felt more recovered 2 h (*P* = 0.014) and 4 h (*P* = 0.026) after LT compared to ST (Table 2).

**Table 2:** **Recovery parameters after both testing sessions including the drop in ΔTSI% during the 6-s sprint, the halftime recovery in ΔTSI% after the 6-s sprint test, and the rate of perceived of exertion 2 and 4 h after the testing session**.

		**Long-track**	**Short-track**
**PPO (W)**			
	Pretest	1244 ± 185	1229 ± 171
	Post-test	1135 ± 179	1096 ± 195
**ΔTSI% 6-S SPRINT**			
Left leg	Pretest	−10.1 ± 4.1	−10.9 ± 6.5
	Post-test	−10.2 ± 4.1	−11.1 ± 6.7
Right leg	Pretest	−10.8 ± 4.0	−11.1 ± 4.6
	Post-test	−10.5 ± 3.4	−12.0 ± 4.9
**HALFTIME RECOVERY ΔTSI% (SEC)**			
Left leg	Pretest	4.7 ± 3.3	4.7 ± 3.8
	Post-test^a^	5.1 ± 3.9	5.9 ± 4.8
Right leg	pretest	4.6 ± 3.7	4.5 ± 3.3
	Post-test^a^	4.8 ± 3.1	5.6 ± 4.0
**RATE OF PERCEIVED EXERTION (1–10)**			
Two hours after^a^		4.2 ± 1.5	5.8 ± 2.0
Four hours after^a^		3.1 ± 1.6	4.6 ± 1.9

a *Significant difference between skating mode (p < 0.05)*.

## Discussion

The aim of this study was to examine the influence of speed skating mode (short-track vs. long-track) on muscle oxygenation in speed skating, and how it consequently affects processes of fatigue and recovery. In order to compare long-track and short-track speed skating, time-trials on both tracks of expected similar physiological intensity were set-up. This was done by asking the participants to perform each time-trial maximally. Although the long-track time-trials were significantly faster and longer in their duration, the lack of difference in heart rate during, and lactate value and rate of perceived exertion directly after the time-trials indicated that both skating modes were of a similar physiological intensity. A comparison of the values for heart rate, lactate, and RPE found in this study with other skating studies indicated that our subjects indeed performed maximally during the time-trials at both tracks (Rundell et al., 1997; Hesford et al., 2013b).

The TT started after a flying lap. The starting values for TSI, tHb, HHb, and HbO_2_ therefore need to be compared to those seen after the first lap of our previous studies which used a standing start (Hesford et al., 2012, 2013a,b). The initial changes seen in our new short-track data are entirely consistent with our previous data (Hesford et al., 2012), i.e., an increase in HHb and a larger fall in HbO_2_ resulting in a drop in TSI and tHb. The amount of hemoglobin in the optical field of view has decreased and what is seen has less oxygen bound. The simplest explanation of this combination of NIRS data is that there is a decrease in blood volume flow and an increase in muscle oxygen consumption. The same trend is seen in the long-track data, indicating that here as well the combination of increased tissue oxygen demand and physical occlusion has caused a combination of vasoconstriction and increased oxygen consumption.

The two skating modes respond similarly after these initial effects. Consistent with our previous studies (Hesford et al., 2012, 2013a,b) there is an increase in muscle oxygenation with time. This is evidenced by a rise in TSI. However, this TSI increase is a result of an increase in HbO_2_ alone. HHb is unaltered with the consequence that tHb increases. The amount of hemoglobin in the optical field of view has now increased and all of this increase is due to hemoglobin that has oxygen bound to it. The rate of oxygen extraction (HHb) does not change with time. The simplest explanation of this data is that the blood volume flow has increased but none of the extra arterial oxygen being delivered is consumed. In effect the blood vessels have expanded but this has not resulted in the muscles consuming more oxygen—the limitation is elsewhere. This does not of course mean that this vasodilation could not be physiologically relevant as it will also increase substrate flow to the muscle and the removal of metabolites.

Where the two skating modes do differ is in their quantitative response to these initial effects, in particular between the asymmetry of the two legs. As shown previously (Hesford et al., 2012), the rate of reoxygenation is faster in the left leg than in the right leg in short-track. However, this is not the case in long-track where no significant differences are seen. At the end if the TT the absolute muscle oxygenation is the same in the right and left legs in long-track and this value is identical to that in the left leg in short-track. However, the right leg in short-track is oxygen deprived compared to these three other states.

The reason for this asymmetry can be seen when exploring the oxygenation changes during a single lap. There are high intramuscular forces on the right leg when traveling around the corner in short-track skating, as the skater hangs on that right leg increasing deoxygenation. Consequently, the left leg that is off the ice, or at least carrying minimal weight, reoxygenates in the corners. In long-track speed skating, the NIRS measurements in combination with video captures showed a cyclic pattern of deoxygenation and reoxygenation during the straights. This pattern is consistent with the repetitive cyclic movement pattern that is executed in long track speed skating, consisting of a gliding phase, a push off phase and a swing phase (De Boer et al., 1987; Allinger and Van den Bogert, 1997; Konings et al., 2015). In the gliding phase and push-off phase, blood flow is occluded and deoxygenation occurs, while in the swing phase reoxygenation occurred. In the corners, a stable and consistent pattern in terms of oxygenation was visible in long-track skating: the difference between the legs is maintained and levels of oxygenation change very little. The movement pattern of leg crossovers in long-track speed skating was rhythmic, cyclic and consistent, and no long on-ice or off-ice phases are seen (Figures 5, 7). Therefore, in long-track deoxygenation largely occurred during the straight not the corners and the changes between the two legs were largely symmetrical. In contrast, in short track speed skating, a very clear asymmetry in reoxygenation occurred during the corners, with the left leg reoxygenating when off the ice and the right leg deoxygenating when on the ice throughout the corner (Figures 6, 8).

Although an asymmetry in muscle oxygenation in long-track speed skating was reported before (Born et al., 2014), the current findings expand on these findings. That study reported that the difference in muscle oxygenation between both legs during the course of a 3000 m TT was only a result of a difference in the initial desaturation between both legs directly after the start (Born et al., 2014). In contrast, the present study shows that the asymmetry between both legs during long-track speed skating is also a result of a slightly enhanced reoxygenation rate in the left leg as the TT progressed (Table 1).

Our results agree with other findings in the literature, relating higher intramuscular pressure to hampered oxygenation and subsequent larger dependency on anaerobic energy supplies, exacerbating fatigue (de Ruiter et al., 2007; Katayama et al., 2007; Romer et al., 2007). In terms of the quantity of differences in muscle oxygenation, we found a reduction of more than 10% in TSI% over the whole test compared to baseline during both skating modes. This is of a similar magnitude to a study in fore-arm muscles, where it was demonstrated that a 7% or larger reduction in muscle oxygenation was found to lead to decreased muscle force production (Murthy et al., 2001).

We hypothesized that the clearly demonstrated differences in deoxygenation and reoxygenation between sports would have an impact on perceived fatigue and recovery, in favor of long-track skating. Indeed, higher experienced fatigue after 2 and 4 h were found after the short-track time-trials compared to the long-track time-trials. This is despite the lack of a difference between lactate values, heart rate data and experienced fatigue when measured directly after the two testing sessions. Some clue to possible mechanisms came from the post-exercise 6-s maximal exercise test. Following short-track skating, there was a clear decrease in the post-exercise reoxygenation rate compared to long-track skating. Intriguingly this difference was seen in both legs, i.e., no differences were found between the right and left legs recovery rates even in short track skating. This might indicate that the differences between both legs are too small to affect processes of recovery after speed skating, however, it might also be a consequence of the relatively long time period (±5 min) between the end of training and the post-test. Nevertheless, whatever the detailed mechanism, the present study provides evidence that short-track speed skating is physiologically more demanding than long track speed skating and that this is associated with a demonstrated difference in hemodynamic patterns both during and post-race and a slower recovery from fatigue.

The application of blood flow restriction in high intensity cycling impacted on lactate accumulation and ratings of perceived exertion (Kim et al., 2015). At low exercise intensities, it was found that RPE was not affected by higher pressures in the range between 40 and 90% of arterial occlusion (Loenneke et al., 2016). A key difference in our skating exercises is that high phases of high intra-muscular pressures in the cycle are alternated with phases of unloading, when the leg is in the swing phase. Alternating pressure patterns and their impact on oxygenation and metabolism in human propulsion have not been studied very often in literature. Our work could inform on the mechanism of Kaatsu training: the application of blood flow restriction during low intensity training that has been shown to produce favorable muscle and vascular adaptations exacerbating training effect. This could be of interest for clinical populations (Loenneke et al., 2016).

In contrast to previous reports that focused on only one skating modality, the current study benefitted from using the same sample of subjects, familiar to both skating modalities, for both long-track and short-track speed skating. In agreement with previous separate studies for short-track (Rundell et al., 1997; Hesford et al., 2012, 2013b) and long-track (Foster et al., 1999), oxygenation changes in the current study are best explained by postulating a reduced blood flow to the working muscles throughout the race for both speed skating disciplines. Most likely, this reduced blood flow caused the relatively high lactate values and low oxygen uptake values in this study. This supports the assumption that a reduced blood flow reduces the aerobic capacity of the recruited muscle groups (Rundell, 1996). The current findings also indicate that the relatively low reported maximal oxygen uptake values in previous long-track speed skating studies (Smith and Roberts, 1991; De Koning et al., 1994) are a consequence of the deoxygenation of the working muscles in speed skating.

In terms of performance, traveling around the corners in short-track skating results in different velocity profiles compared to long-track speed skating. The subjects were slower during short-track speed skating when they traveled around the corner standing at one leg only. To regain their loss in velocity, the subjects accelerated when they came out of the corner and during the straight. Consequently, short-track seems to show more variability in velocity in each lap, while the long-track velocity profile seems more continuous (see Figure 1). Fluctuations in velocity were larger in short-track speed skating, and large fluctuations around the mean velocity are associated with higher aerodynamic energy losses (Van Ingen Schenau et al., 1983; De Koning et al., 1999; Hettinga et al., 2011, 2012). In running, a more variable interval training pattern has been shown to be associated with a slower recovery than demonstrated after continuous training (Townsend et al., 2013). In that sense, this finding also confirms that short-track speed skating is the more demanding sports of the two, and might need a longer recovery after exercise and training.

For trainers and coaches it is important to realize that due to this element of occlusion and reduced blood flow as well as the larger fluctuations in speed, physiological load is higher than expected for their athletes during short-track speed skating compared to long-track speed skating. Moreover, training schedules based on physiological variables achieved during other non-occluded sports, such as commonly used (maximal) cycling tests, might not represent the actual physiological load of the training sessions, and trainers should beware of risks of overtraining. The working muscles of the right leg remained relatively more deoxygenated during short-track compared to long-track speed skating. This indicates that the muscles in short-track speed skating are more anaerobic than in long-track speed skating. However, as oxygen extraction does not change with time even when oxygenation recovers in long-track skating, it is not clear that this has adverse metabolic consequences at least in terms of oxygen metabolism. Nevertheless, the present study shows that a short-track training session leads to higher experienced fatigue in the hours after the training session and seems to need a longer recovery period compared to a similar long-track session.

Our findings emphasize the importance of pacing in speed skating. In particular the ability to maintain the optimal technical skating characteristics (respectively, small knee-, trunk-, and push-off angle) during the whole race, despite processes of fatigue, seemed to be crucial in elite (long-track) speed skating (Hettinga et al., 2011) and lead to different pacing behavior in speed skating compared to for example cycling (Stoter et al., 2016). Less explosive pacing strategies are chosen in speed skating, to make sure not to collapse at the end of the race. The present study showed that the crouched skating position indeed seemed to lead to a physiological disadvantage: the deoxygenation of the working muscles, which may cause an earlier onset of fatigue. As in the short-track time-trials in the present study, a relatively high decrease in velocity toward the end of the race was demonstrated, also in short-track speed skating the ability to maintain optimal technical skating characteristics at the end of the race seems crucial (Konings et al., 2016). In particular in actual competition this will be of importance, as this is performed in heats, competing against opponents (Konings et al., 2016; Noorbergen et al., 2016).

In conclusion, the current study showed that the patterns of reoxygenation and deoxygenation in the working muscles during a race are different for long-track and short-track speed skating. This seems to be a result of the high intramuscular forces on the right leg when traveling around the corner in short-track skating: athletes are usually standing on the right leg throughout the corner, that thereby remains deoxygenated for a long period where the left leg reoxygenates in the same phase when off the ice. In long-track skating, a more symmetric profile was demonstrated, in which over the straights the cyclic pattern of stance phase, push off phase and swing phase was clearly visible in terms of oxygenation. In the corners, the rhythmic leg crossovers resulted in a rather stable value for deoxygenation, that was larger for the right leg compared to the left leg. The results demonstrated that short-track speed skating seems to be the more physiologically demanding sport. This subsequently led to higher experienced fatigue directly after the exercise, as well as to longer periods of recovery needed in the hours after the short-track compared to long-track speed skating. These results can help coaches and athletes in optimizing their training protocols, and provide us with more insights into the mechanistic physiological principles relevant for elite athletes in different sports, and on how technical factors are impacting on those.

## Author contributions

The measurements were conducted at InnoSportLab Thialf, with portable equipment available from the laboratory of the University of Essex. FH, MK, and CC all contributed to conception and design of the work. MK collected data. FH, MK, and CC were involved in analysis and interpretation. MK, FH, and CC wrote the paper. All authors have approved the final version of the manuscript, agree to be accountable for all aspects of the work in ensuring that questions related to the accuracy or integrity of any part of the work are appropriately investigated and resolved, and all persons designated as authors qualify for authorship, and all those who qualify for authorship are listed.

## Funding

The work described here was funded in part by grant EP/F005733/1 from the UK Engineering and Physical Sciences Research Council (EPSRC). There were no additional funding sources for this study. The funders had no role in study design, data collection and analysis, decision to publish, or preparation of the manuscript.

### Conflict of interest statement

The authors declare that the research was conducted in the absence of any commercial or financial relationships that could be construed as a potential conflict of interest.
